# Directed evolution to improve protein folding *in vivo*

**DOI:** 10.1016/j.sbi.2017.12.003

**Published:** 2017-12-23

**Authors:** Veronika Sachsenhauser, James CA Bardwell

**Affiliations:** 1 Department of Molecular, Cellular and Developmental Biology, University of Michigan, 830 N. University, Ann Arbor, MI 48109, USA; 2 Howard Hughes Medical Institute, University of Michigan, 830 N. University, Ann Arbor, MI 48109, USA

## Abstract

Recently, several innovative approaches have been developed that allow one to directly screen or select for improved protein folding in the cellular context. These methods have the potential of not just leading to a better understanding of the *in vivo* folding process, they may also allow for improved production of proteins of biotechnological interest.

## Introduction

Most proteins are only marginally stable, exhibiting net free energies of folding in the range of only a few kcal/mol [1,2^••^]. This marginal stability is not only a source of experimental frustration to scientists working with proteins but also greatly inhibits the use of proteins for biotechnological purposes. Optimizing protein folding would thus be of great practical value. Moreover, *in vivo* optimization of folding would give us a better understanding of how proteins normally fold in the cell, possibly granting us insight into how protein misfolding can lead to disease. Improving protein stability either *in vivo* or *in vitro*, however, is rather challenging as most amino acid substitutions are destabilizing [1], and those rare stabilizing variants that can be found often interfere with protein function [1,3,4]. Attempts to circumvent this function-stability tradeoff by manipulating cellular chaperones in beneficial ways is also challenging; this is not surprising as millions of years of evolution have been at work optimizing these *in vivo* folding machines. Most chaperones are designed to work on many proteins [5]. This fact may help explain why chaperone variants specifically selected for improved function with one protein often show impaired function in their folding of other proteins [6]. These issues may help account for the mixed results that have been obtained with protein stabilization efforts.

Although our general understanding of protein folding has been significantly advanced by *in vitro* experiments, applying the lessons learned *in vitro* to improve folding *in vivo* has been difficult. *In vivo* folding differs substantially from *in vitro* folding due to the effects of macromolecular crowding, hindered diffusion, co-translational folding, and chaperone-facilitated folding that are at play in the cellular environment. These areas have been the subject of recent excellent reviews and so will not be further discussed here [7–10]. Innovative in-cell reporting systems that allow for the fluorescent detection of *in vivo* protein denaturation after in-cell urea titration suggest that at least protein thermodynamic stability is not radically different in the *in vivo* and *in vitro* environments [11,12^••^,^13^]. However, *in vivo* stability is not just thermodynamic stability; *in vivo* stability also entails the protein’s persistence in a functional, non-aggregated form in the cell. While *in vivo* protease susceptibility is roughly correlated with *in vitro* thermodynamic stability [2^••^,^14^], aggregation susceptibility is poorly assessable as it is strongly affected by the *in vivo* environment. Aggregation is a crucial factor for rationally designed proteins, as proteins often fold into insoluble oligomeric states *in vivo* due to the effect of unanticipated intermolecular interactions that occur within the cell [15].

Optimizing protein folding in the cell could at least in principle fix many of these problems. The simplest and most generic methods commonly used to improve protein folding *in vivo* are by optimizing growth and expression conditions, including growth temperature, time of induction, promoter strength, inducer concentration, codon usage, and the use of solubility-enhancing fusion tags. These approaches are well documented and will thus not be discussed further here [16–19]. A more targeted way to improve protein folding is by using directed evolution. These methods typically involve the generation of a pool of mutant variants followed by a selection process to find those with improved folding properties. Through multiple iterative rounds of Darwinian selection, folding-optimized variants with multiple mutations can be obtained, including many that are unlikely to have been generated using rational design or phylogenetic comparison approaches. In addition to evolving the proteins themselves for improved stability, directed evolution can also be used to customize host organisms to provide an optimized folding environment for specific proteins [20].

In this review, we outline recent advances in harnessing the power of directed evolution to optimize protein folding in the cell. Approaches include novel selection and screening methods for protein variants and host strains as well as the evolution of chaperones.

## Harnessing the power of molecular biology for genetic diversification

Genetic variation can be generated using several different methods. Classic random mutagenesis techniques such as chemical and physical mutagenesis, and error prone PCR, transposon insertion mutagenesis, gene shuffling as well as more recently developed technologies for targeted mutagenesis including Multiplex Automated Genome Engineering (MAGE) facilitate the introduction of genetic changes *in vitro* and *in vivo*. The diverse methods are described in several excellent reviews and articles and will not be discussed further here [21–24].

## Systems to assay for improved protein folding

**Figure 1:**
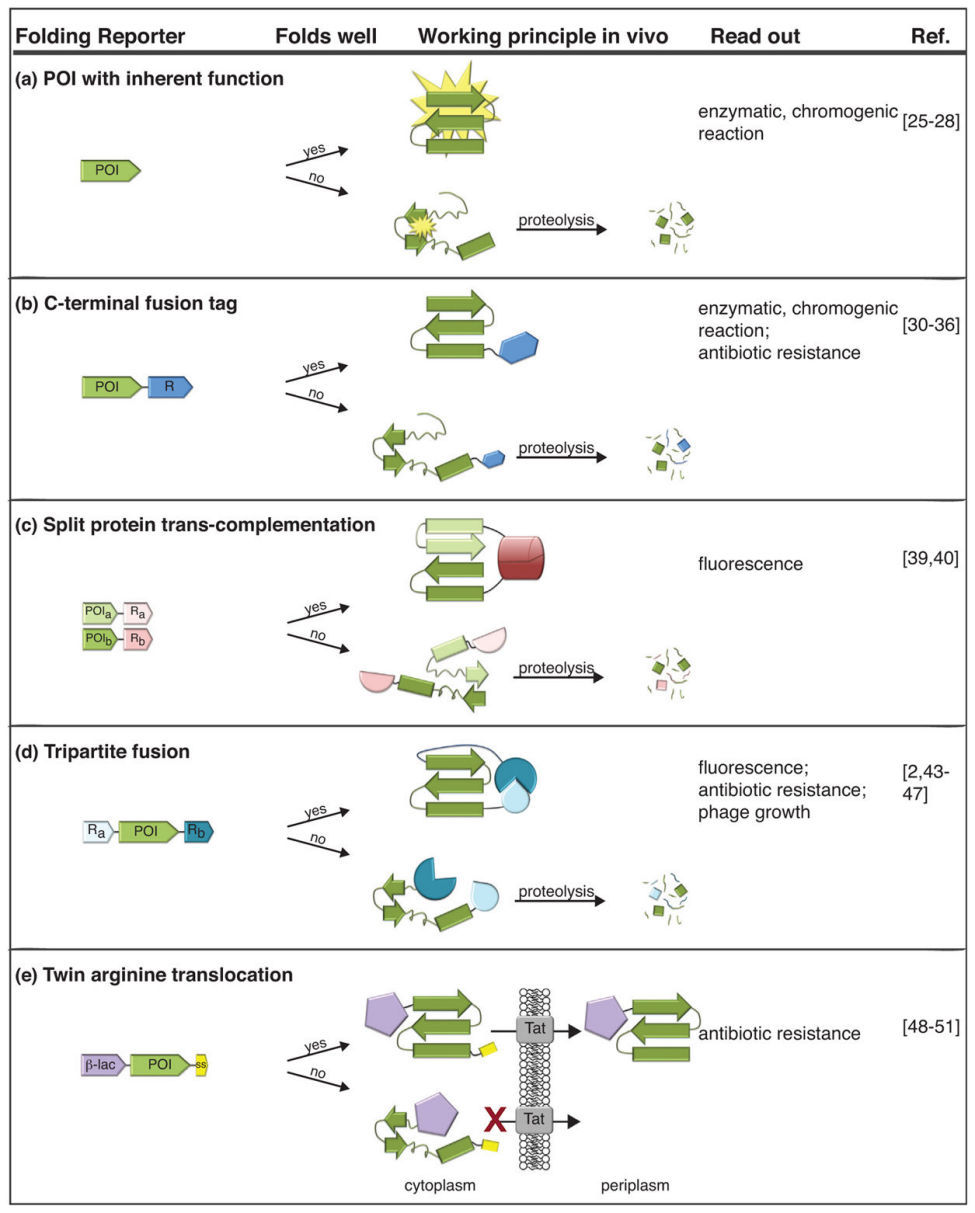
Selection and screening systems for improved protein folding *in vivo*. **(a)** An inherent, measurable function (e.g. enzymatic, chromogenic reaction) of a protein of interest (POI) is exploited to screen for stabilized protein variants. **(b)** A screen or selection for stabilized POI variants is enabled by fusion to a reporter protein with a measurable function. **(c)** Tight interaction of two portions of POI variants leads to proper complementation of fused fluorescent reporter protein portions and thereby allows screening for stabilized protein variants. Unstable protein variants will be depleted from the screen by degradation. **(d)** Split halves of a reporter protein will only interact and confer its inherent selectable/screenable function if the inserted protein variant folds well and doesn’t get proteolyzed or aggregated. **(e)** The twin arginine translocation (Tat) system relies on translocation of exclusively well-folded protein variants into the periplasm where proper folding is additionally selected for by a fused β-lactamase tag. POI, protein of interest; POI_a_/POI_b_, POI_a_ half or POI_b_ half, respectively, of split POI; R_a_/R_b_, R_a_ half or R_b_ half, respectively, of split reporter protein R; P, proteolysis; Tat, twin arginine translocase; ss, Tat signal sequence; β-lac, β-lactamase resistance marker.

The accuracy, power, throughput, and stringency of selection or screening approaches are crucial to the success of a Darwinian optimization process. Various selection and screening systems have recently been developed to allow for the identification of stabilized protein variants (Figure 1).

## Selection for improved folding based on an endogenous property

One very straightforward way to select for increased stability is to take advantage of an inherent property of the protein of interest, such as its enzymatic activity or any other property that can be easily screened for (Figure 1a). Unfortunately, the limited availability of simple assays for these properties makes this approach very protein-specific and generally relatively low-throughput [25–28]. However, one recent and very promising approach [29^••^] allows one to directly assay protein solubility *in vivo*, bypassing the need for protein-specific assays. In this assay, cells expressing the protein are incubated at elevated temperatures, lysed on a Durapore membrane filter which blocks variants that tend to aggregate but allows variants that remain soluble to pass through. These soluble variants are then retained on a nitrocellulose membrane and detected either with antibodies against the protein of interest itself or against an affinity tag attached to the protein. This method is not protein-specific, thus potentially broadly applicable and can be conducted in high-throughput.

## Improving protein folding using folding reporter tags

Another way around the lack of an easily assayed property is to fuse the protein of interest to a reporter protein with the hope that the activity of the reporter will reflect the folding of its fusion partner. Improved folding of a tagged protein variant, for instance, could result in a parallel increase in the amount of the reporter protein. Quantification of the reporter then provides an indirect measure of the effective abundance of the protein of interest (Figure 1b). Green fluorescent protein (GFP) is one of the first and most commonly used fusion tags employed in screens for improved stability *in vivo* [30,31]. By combining GFP fusions with fluorescence activated cell sorting (FACS), folding variants can be screened and isolated in high throughput [32]. Other C-terminal reporter fusion tags that rely on chromogenic, enzymatic, or antibiotic resistance-conferring properties have also been developed and applied to screen or select for improved folding [33–36]. For example, dihydrofolate reductase has been used as a solubility reporter to select for soluble expression constructs [37]. These reporter fusion tag systems have disadvantages, however. They entail a propensity for false positives due to truncations or cleavage artifacts and fusions can alter the solubility of the protein variant [38].

To help eliminate these problems, split GFP systems have been developed in which only a short, non-fluorescent portion of GFP is fused to the C terminus of the protein of interest (Figure 1c). Expression of the remainder of GFP from a second plasmid will only complement the short portion well (and thereby emit fluorescence) if the protein of interest is soluble and remains intact (i.e. it is not degraded). The short nature of the GFP portion of these fusion constructs links it more closely to the folding and solubility properties of the protein of interest [39^•^].

In an alternative approach, Lindman *et al.* successfully used trans-complementation of fragments of GFP to screen for improved thermodynamic stabilization of the B1 domain of protein G (PGB1) [40,41^•^]. The rationale here is that mutations that stabilize a protein chain, in this case of PGB1, will tend to increase the affinity between two fragments of that chain. If these two fragments are fused to different portions of GFP, fragment stabilization will tend to drive the GFP portions together, resulting in better complementation and increased fluorescence [41^•^].

## Tripartite protein folding sensors to optimize protein stability

More recently, advanced tripartite folding reporters have been developed in which a protein of interest is inserted at a permissive site within a reporter protein (Figure 1d). In the Proside (Protein stability increased by directed evolution) approach, the protein is inserted between two domains of an essential bacteriophage capsid protein. Stable variants will be more resistant to *in vitro* proteolysis and can be selected on that basis [42,43]. Several *in vivo* folding biosensors have also been developed based on a similar rationale; that is, the two parts of the reporter protein will only be able to fold together and confer the reporter’s intrinsic function if the inserted protein folds well. If the inserted protein is poorly folded, it will be cleaved by the plethora of proteases in the cell. This will separate the two halves of the reporter, resulting in lower levels of reporter function. This cis-complementation tripartite fusion approach is advantageous in that it discriminates against artifacts arising from internal ribosome initiation sites and other events that can untether the reporter from the target protein. Several tripartite protein folding reporter systems have been developed based on GFP [44]. Tripartite protein-based systems that rely on antibiotic resistance markers enable an efficient ‘fold or die’ selection for improved stability [2^••^,^45^]. The protein of interest is fused between the split marker halves of an antibiotic resistance gene. Improved folding of the inserted protein will result in complementation of the split marker halves and in turn, increased antibiotic resistance. Foit *et al*. optimized folding of Immunity protein 7 (Im7) in the *Escherichia coli* periplasm by applying a β-lactamase-based tripartite system. Interestingly, mutants that enhanced the thermodynamic stability of Im7 almost entirely mapped to surface residues involved in binding to its natural binding partner E7, suggesting that a stability-function tradeoff exists for this protein. This β-lactamase-based tripartite system has been applied to evaluate and further evolve the folding of rationally designed proteins [46,47] and to identify small molecule inhibitors of aggregation *in vivo* [48^•^].

Another tripartite selection also couples proper folding of a test protein to antibiotic resistance but is based on an entirely different principle: only folded proteins will be efficiently exported to the periplasm by the twin arginine transport (Tat) quality control system (Figure 1e). In this approach [49,50,51^••^], the Tat signal sequence is fused to the N-terminus of the protein of interest followed by fusion to β-lactamase, which will only encode antibiotic resistance if it is exported into the periplasm. The test protein must be properly folded to be recognized and exported by the Tat apparatus. This selection has been used to improve the *in vivo* solubility of several proteins [49,50,52,53]. A two-hybrid type version of this approach, based on the ability of the Tat translocase to carry with it non-covalently interacting proteins, has enabled the selection of protein variants with stronger protein-protein interactions [54–56] and enhanced intracellular stability [57].

## Evolving an improved folding environment

Protein folding is not exclusively dependent on the protein sequence — the cellular folding environment is also important. There has been significant interest in engineering bacterial strains for the improved folding and expression of recombinant proteins [58]. Folding can be optimized by evolving the redox capacity to facilitate disulfide bond isomerization [59,60]. A specially designed strain called SHuffle has been generated that contains several cleverly targeted alterations and shows substantially improved folding of proteins with multiple disulfide bonds [61^•^].

General chaperone overexpression can promote *in vivo* protein folding [56]. It fosters protein evolution through buffering the destabilizing effect of thermodynamically unfavorable mutations of folding intermediates [58,62,63]. However, this approach is not aimed at improving the folding of specific proteins and is not broadly applicable given that even the generalist chaperone GroEL is estimated to interact with only about 10% of proteins in *E. coli* [64,65]. Chaperone expression has already been efficiently balanced in the cell by evolution, perhaps explaining why chaperone co-expression is only occasionally successful in improving the *in vivo* expression of specific proteins [66,67].

A more effective approach to improve *in vivo* folding may be to specifically evolve the cellular environment for the folding of a single protein of interest. Our group tested this strategy by using the tripartite β-lactamase approach in *E. coli* to select for host variants that improved the folding of Im7 [68]. The selected variants overproduced the periplasmic chaperone Spy. This protein, when purified, was shown to inhibit the aggregation and facilitate the refolding of a variety of proteins including Im7.

As protein folding can be improved by overexpressing client-specific chaperones, expression of a specifically optimized chaperone should have a similarly beneficial effect. It appears that the foldability of proteins and the sequence of highly specific chaperones has co-evolved [69]. Thus, it seems unlikely that these types of chaperone–client interactions can be easily further optimized. In contrast, promiscuous chaperones that normally interact with many different binding partners can be evolved to enhance their interaction with one specific client protein. A detailed review about chaperone enhancement has been published recently by Mack *et al*. [70].

For example, Wang *et al*. evolved variants of the Hsp60 chaperone GroEL and co-chaperone GroES that showed an enhanced ability to stabilize GFP in *E. coli* [6]. Unfortunately, these GroEL/S mutants were defective in their ability to fold other proteins, reflecting the specificity-promiscuity tradeoff that one is faced with in the evolution of most chaperones.

Aponte *et al*. focused on improving another chaperone, Hsp70 DnaK. The evolved chaperone showed several fold improved refolding ability for soluble, denatured luciferase as compared to wild-type DnaK [71]. Their selection system was based on a destabilized antibiotic resistance marker that only confers antibiotic resistance *in vivo* if properly folded (a variation on the schema illustrated in Figure 1a). Overexpression of effective DnaK variants allowed the phenotype to be rescued.

Our group has isolated Spy variants that not only improve the stability of unstable Im7 mutants, but other proteins as well, implying that they may be generally more effective [72^•^,^73^,^74^]. One interesting class appears to work by enhancing the flexibility of a segment in Spy whose flexibility is known to be important for Spy’s action. Interestingly, the residue change found in one of these ‘super Spy’ mutants is actually quite common in evolution. It seems likely that the affected residue may act as an evolutionary rheostat, tuning the flexibility of this segment to fit the organism’s need for Spy’s substrate diversity, balanced with its need to maintain some minimal stability for Spy.

A comparable concept has been observed with potentiated Hsp104 variants selected for increased disaggregation activity. In this case as well, some mutants appear to act by increasing the flexibility of the chaperone [75], highlighting the importance of chaperone flexibility in their action [76].

## Conclusions and outlook

Insights from directed evolution studies highlight the various and sometimes unexpected ways that protein folding, the product of many years of evolution, can actually be improved upon *in vivo*. Newly developed fusion approaches that allow for the direct selection of proteins with improved folding *in vivo* and direct ways of screening for improved solubility present a diverse platform for future creative endeavors.
